# Maturity onset diabetes of the young and fibrin-related thrombosis risk

**DOI:** 10.1177/1479164120963048

**Published:** 2020-12-18

**Authors:** RC Sagar, F Phoenix, G Thanabalasingham, K Naseem, RA Ajjan, KR Owen

**Affiliations:** 1 Leeds Institute of Cardiovascular and Metabolic Medicine, Faculty of Medicine and Health, University of Leeds, Leeds, West Yorkshire, UK; 2 Oxford Centre for Diabetes, Endocrinology and Metabolism, University of Oxford, Oxford, UK; 3 Oxford NIHR Biomedical Research Centre, Churchill Hospital, Oxford, UK

**Keywords:** MODY, fibrinolysis, thrombosis, clot structure

## Abstract

**Background:**

Fibrin network characteristics determine predisposition to cardiovascular disease (CVD). Individuals with type 1 (T1DM) and type 2 diabetes mellitus (T2DM) have higher risk of CVD and display deranged fibrin network structure. Those with maturity onset diabetes of the young (MODY) may also be at increased risk but their fibrin clot properties have not been studied.

**Methods:**

Plasma clots properties from 13 individuals with HNF1A-MODY, 12 matched-individuals with T2DM and 12 with T1DM were studied using a validated turbidimetric assay and confocal microscopy. Plasma levels of fibrinogen, plasminogen activator inhibitor-1, complement C3 and C-reactive protein were also measured.

**Results:**

MODY clot maximum absorbance was 0.37 ± 0.03 AU, similar to T1DM (0.32 ± 0.03 AU; *p* = 0.26), but lower than T2DM (0.49 ± 0.03 AU; *p* = 0.02), with confocal microscopy confirming structural differences. Clot lysis time in MODY was similar to T1DM (456 ± 50 and 402 ± 20 s, respectively; *p* = 0.09) but shorter than T2DM (588 ± 58 s; *p* = 0.006). Comparing inflammatory/thrombotic proteins in HNF1A-MODY and T2DM, C3 levels were lower in MODY than T2DM (0.58 ± 0.09 and 0.80 ± 0.1 mg/ml, respectively; *p* < 0.01).

**Conclusions:**

HNF1A-MODY fibrin network alterations are at least as pronounced as in T1DM but less thrombotic than T2DM clots. Differences in fibrin clot characteristics comparing HNF1A-MODY and T2DM may, in part, relate to lower C3 levels.

## Introduction

Whilst cardiovascular complications are recognised as significant contributors to morbidity and mortality in patients with both type 1 (T1DM) and type 2 diabetes (T2DM),^1,2^ far less is known about the risk in maturity onset diabetes of the young (MODY). MODY is group of monogenic disorders of β-cell function, leading to reduced insulin secretion and diabetes with a predominantly pancreatic phenotype.^3,4^ It is estimated that MODY is the cause of around 2% of patients with diabetes, although this may be as high as 5%, particularly as the diagnosis is often delayed or missed.^4^ Whilst the percentage of individuals with MODY is relatively small, the actual number of these patients is significant with a prevalence of at least 1 in 10,000 in the adult population. Despite this, little is known about the cardiovascular risk in these patients and how their outcomes compare with macrovascular complications in both type 1 and type 2 diabetes. Limited studies suggest that risk of vascular complications is increased in individuals with MODY compared to individuals without diabetes except for those with glucokinase gene mutation who display no or minimal increase in risk.^5^ A Finnish cohort of HNF1A-MODY had similar levels of microvascular complications to T1DM and T2DM when matched for diabetes duration and glucose control assessed as glycated haemoglobin (HbA1c). The prevalence of coronary artery disease (CAD) appeared to be midway between T1DM and T2DM, although the T2DM group were significantly older.^6^ In another study, Irish patients with HNF1A-MODY had the same prevalence of CAD as a matched T1DM group.^7^

We have previously shown that fibrin clot properties, in particular fibrin clot lysis, predicts vascular risk and clinical outcome in individuals with diabetes.^8,9^ In this pilot study we hypothesise that clot structure is altered in individuals with the commonest form of MODY, due to pathogenic variants in *HNF1A*, which in turn may be one mechanism for the enhanced risk of vascular complications in this population. Therefore, in this work we compare clot structure parameters and key coagulation protein plasma levels in individuals with HNF1A-MODY to those with T1DM and T2DM, groups with known increased vascular risk compared to the healthy population.^10,11^

## Materials and methods

For the study, 13 volunteers with known MODY due to an *HNF1A* variant from the MODY in Oxford Study (REC A 06/Q1604/136) were studied. Twelve individuals each with T2DM and T1DM, matched for age and HbA1c, were also recruited. The individuals with T2DM were recruited from the Young Diabetes in Oxford study (REC A 04 Q1604 97) and individuals with T1DM from Leeds (REC 09 H1307 12). After informed consent, morning blood samples were collected in sodium citrate (3.8%) and centrifuged within 2 h of collection to separate plasma. All plasma samples were stored at –80°C until required for analysis. Clinical data were also recorded.

Clot structure was studied in all these samples using a validated turbidimetric assay as previously described.^12,13^ Clot final turbidity, used as a measure of clot density, was recorded. We also investigated time from full clot formation to 50% lysis as a marker of lysis potential as well as clot lysis area, a complex measure of clot formation and lysis. Clots generated *ex vivo* were visualised using confocal microscopy, as previously described elsewhere.^14^ Plasma levels of fibrinogen and plasminogen activator inhibitor-1 (PAI-1) along with inflammatory proteins, C-reactive protein (CRP) and complement-3 (C3) were analysed by enzyme-linked immunosorbent assay (ELISA) as previously described.^8,15,16^

All statistical analyses were conducted using PRISM 8.3.1. ANOVA was used comparing the three groups with unpaired *t*-test for comparing two groups.

## Results

Our baseline characteristics are outlined in Table 1 and shows that the groups were well matched for age, gender, BMI and HbA1c.

**Table 1.** Clinical characteristics of the three patient groups.

	T1DM (*n* = 12)	MODY3 (*n* = 13)	T2DM (*n* = 12)	*p* value
Mean age (range)	26 (19–41)	36 (19–51)	36 (21–52)	0.96
Male/Female	6/6	6/7	5/7	0.68
BMI	25.5 ± 1.6	26.3 ± 1.6	27.6 ± 2.2	0.71
HbA1c (%)	7.8 ± 0.3	7.1 ± 0.4	7.6 ± 0.5	0.44
Sys BP (mmHg)	133 ± 5	131 ± 7	122 ± 5	0.30
Dias BP (mmHg)	73 ± 3	77 ± 4	77 ± 3	0.74
Smoker (%)	17%	18%	0%	0.44
On statin (%)	8%	23%	42%	0.16
On aspirin (%)	0%	8%	8%	0.60
On insulin (%)	100%	39%	50%	0.004

T1DM: type 1 diabetes, MODY: maturity onset diabetes of the young, T2DM: type 2 diabetes, BMI: body mass index, Sys BP: systolic blood pressure, Dias BP: diastolic blood pressure.

Values given as mean ± SEM or percentages.

### Clot maximum absorbance (final turbidity) and lysis time

**Figure 1.**
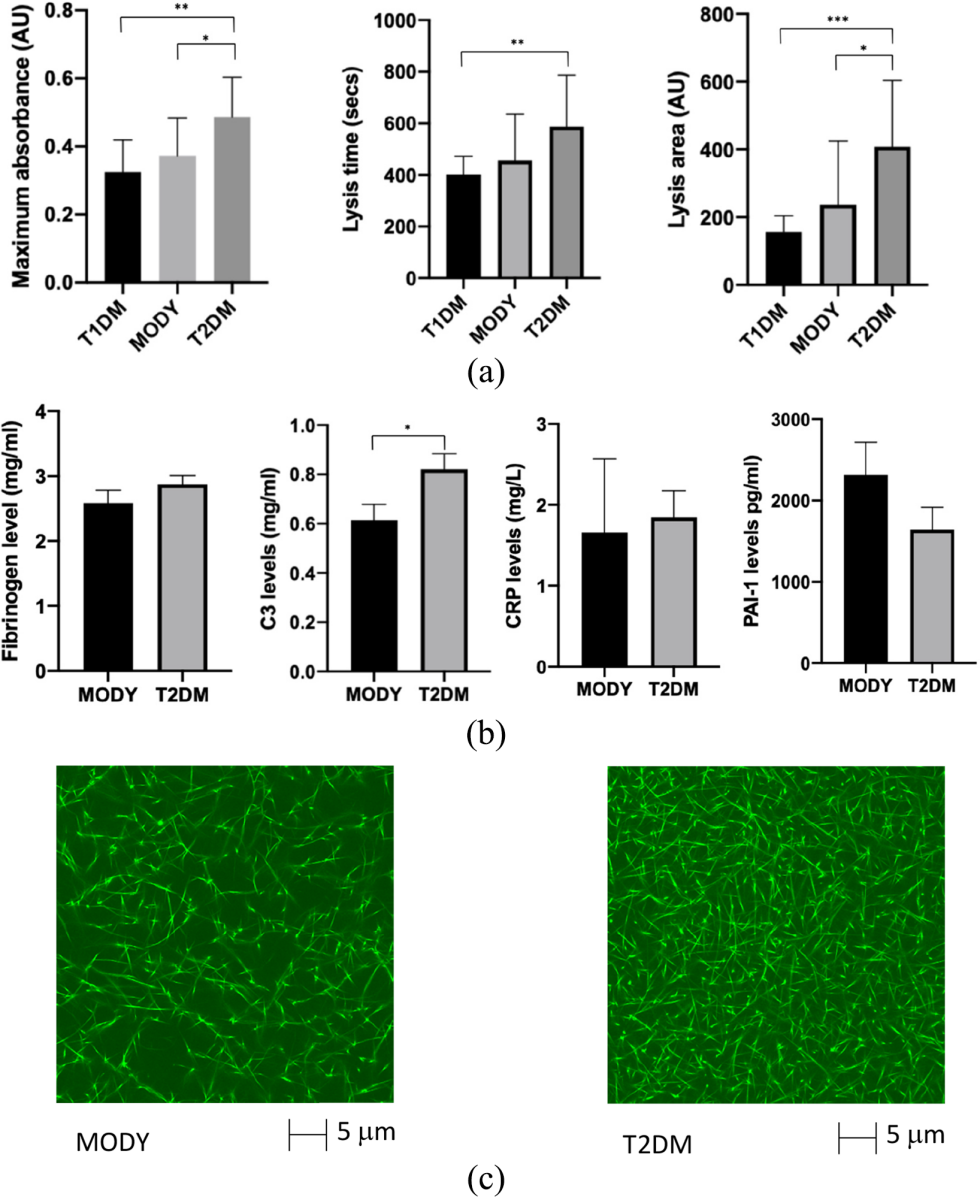
Fibrin clot characteristics of clots made from plasma samples in maturity onset diabetes of the young (MODY, *n* = 13), Type 1 diabetes (T1DM, *n* = 12) and Type 2 diabetes (T2DM, *n* = 12). (a) Clot maximum absorbance, lysis time and clot lysis area. (b) Plasma levels of thrombotic and inflammatory proteins, including fibrinogen, plasminogen activator inhibitor (PAI)-1, C-reactive protein (CRP) and complement C3. (c) Confocal microscopy of pooled plasma samples from MODY and T2DM groups.

There were significant differences seen in clot maximum absorbance between the three groups (*p* = 0.003). The T1DM had the lowest maximum absorbance, 0.32 ± 0.03 AU, which was not significantly different from the MODY group, 0.37 ± 0.03 AU (*p* = 0.26). In contrast T2DM plasma had a clot maximum absorbance of 0.49 ± 0.03 AU, significantly higher than both the T1DM and MODY groups, *p* = 0.001 and *p* = 0.02, respectively (Figure 1(a)). Similarly, lysis time was found to highest in the T2DM group (588 ± 58 s) with lower times observed in MODY and T1DM (456 ± 50 and 402 ± 20, respectively).

### Clot lysis area

Clot lysis area is a measure reflecting both clot formation and lysis, and has previously been used to predict the risk of vascular events.^13^ The T1D group had the smallest lysis area (157 ± 14 AU) followed by the MODY group (238 ± 52 AU (*p* = 0.16)). The largest lysis time was observed with T2DM (409 ± 56 AU), which was significantly elevated compared to T1DM (*p* < 0.01) and MODY (*p* = 0.04). The differences seen between the three groups was also statistically significant, *p* = 0.002 as shown in Figure 1(a).

### Thrombotic and inflammatory proteins

Our data have so far shown that plasma clot structure from individuals with MODY is largely similar to that of T1DM, but significantly different compared with T2DM. To understand potential mechanisms for the observed differences between MODY and T2DM groups, we next examined plasma levels of key thrombotic and inflammatory proteins. Fibrinogen, plasminogen activator inhibitor (PAI)-1 and C-reactive protein (CRP) levels were similar when comparing the MODY and T2DM groups. In contrast, complement-3 (C3) levels, an inflammatory protein with antifibrinolytic properties, were lower in the MODY group compared with T2DM (0.58 ± 0.09 vs 0.80 ± 0.1, *p* < 0.01; Figure 1(b)).

### Confocal imaging

We next imaged the clot structure in pooled samples from the MODY and T2DM groups. The images demonstrate a tighter plasma clot structure from T2DM plasma compared with plasma (Figure 1(c)), consistent with the differences observed on turbidimetric assays.

## Conclusion

Our data suggest that individuals with HNF1A-MODY have a prothrombotic plasma clot structure that is at least similar to those with T1DM but it differs significantly compared to individuals with T2DM. It can be argued that MODY patients display an intermediate fibrin structure phenotype, sitting between T1DM and T2DM individuals, supported by data from clot lysis area but numbers are limited to make concrete conclusions. Taken together, our findings indicate increased thrombosis risk in MODY that is likely to contribute to their vascular risk.

Given the clear differences observed between MODY and T2DM patients, we investigated potential mechanisms and demonstrate this is not related to fibrinogen or PAI-1 plasma levels. Interestingly, C3 plasma levels were found to be significantly lower in MODY compared with T2DM. Given the possible effects of C3 on clot structure and fibrinolysis, whereby it binds to fibrin and is incorporated into the clot resulting in prolonged fibrinolysis^16^ as well as the role of HNF1A-MODY in modulation of terminal complement proteins,^17^ this may be one mechanism by which to explain the differences seen between our patients with HNF1A-MODY and those with T2DM. Alternatively, it is possible that HNF1A-MODY patients display alternative post-translational modification of plasma proteins, particularly fibrinogen, which may account for the observed differences in fibrin network structure, measured by the turbidimetric assay and visualised by confocal microscopy.

The main strengths of the study are the investigation of clot structure in HNF1A-MODY, which has not been undertaken before and the well-matched groups of patients. Also, we used a validated turbidimetric assay to study clot structure and further visualised the clot by confocal microscopy to confirm the findings. In addition, our data suggest that reduction of complement C3 levels may be a protective mechanism in HNF1A-MODY against the formation of more thrombotic clots.

The main limitation to our study is the relatively small cohort of subjects involved. The findings are therefore preliminary, but could point to differences between the diabetes types. As a cross-sectional study, causality (i.e. clot structure contributing to vascular events) was not measured, but our evidence suggests prospective outcome studies are warranted. More work is needed to understand the mechanistic pathways responsible for the differences in clot structure parameters comparing HNF1A-MODY and T2DM. HNF1A is a transcription factor, regulating expression of a large number of hepatic genes including the key clotting molecules prothrombin, fibrinogen as well as the terminal clotting factors C5 and C8. This may lead to unique effects on clot formation in HNF1A-MODY. Finally, as we only studied one type of MODY, our observations may not be generalisable to other forms of monogenic diabetes.

In summary, we demonstrate that fibrin-related thrombosis risk in MODY is at least as high as in T1DM, but does not reach the risk level seen in T2DM. Our work indicates that further research, with a greater number of participants, is warranted into evaluating fibrin network properties in MODY and how this contributes to vascular outcome. Studies are also required to analyse other types of MODY and mechanistic pathways responsible for the observed changes in fibrin network structure, which may offer novel treatment strategies to reduce the risk of thrombosis in this population.
